# SELF-RATINGS OF HEALTH BY AGE AND BIRTH COHORT: FOR WHOM DO THEY PREDICT MORTALITY?

**DOI:** 10.1093/geroni/igad104.1406

**Published:** 2023-12-21

**Authors:** Ellen Idler, Emily Dore

**Affiliations:** Emory University, Atlanta, Georgia, United States; Emory University, Atlanta, Georgia, United States

## Abstract

Global self-rated health as a predictor of mortality has become one of the most reliable outcomes in population-based follow-up studies. However, few studies focus on age or birth cohort differences in the mortality predictiveness of self-ratings of health. Personal knowledge of health accumulates throughout the life course, leading to the hypothesis that self-rated health would be most predictive in old age or in earlier birth cohorts. However, the pronounced effect of self-rated health on mortality means that those with the higher mortality risk of poor self-rated health in earlier adulthood would be less likely to survive to old age, potentially diminishing the effect through selective survival. In this paper we test for potential variations in the effects of age and birth cohort on the association between self-rated health and mortality in a large sample of adults aged 25-101 in the Panel Study of Income Dynamics (N=6437) followed from 1999-2019. We ask if self-rated health -- net of sociodemographic factors, physical and mental health conditions, and health risk behaviors – is equally predictive of mortality in three age groups spanning young, middle, and late adulthood, and in four birth cohorts. Fully adjusted models show that self-rated health was the most predictive for the group aged 41-64, and for those born 1946-1980, and that there were nearly null effects for those aged 25-40 or born 1981-1994. Thus, the effects of self-rated health on mortality vary by age and cohort, in ways that support both knowledge-based and selective survival interpretations of the association.

